# Supplementary material for: Using adopted individuals to partition indirect maternal genetic effects into prenatal and postnatal effects on offspring phenotypes
Source: eLife. 2022 Jul 13;11:e73671. doi: 10.7554/eLife.73671 (PMC9323003; doi:10.7554/eLife.73671)
Supplement: Supplementary file 6. — Source Code File. R scripts that fit Structural Equation Modelling to partition maternal genetic effects into pre- and postnatal effects on offspring phenotypes. [file elife-73671-supp6.docx]

**Supplementary File 6.** Some limitations/assumptions of our Structural Equation Model (SEM) and its application to the UK Biobank.

| **Description of assumption/limitation** | **Comments** | **Consequences on model parameters** | **Possibilities for addressing the limitation** |
| --- | --- | --- | --- |
| 1. Limitations/Assumptions specific to the UK Biobank dataset (i.e. limitations/assumptions that are largely a consequence of working with adopted singletons in a cohort that was not set up to examine adopted individuals specifically) | | | |
| Paternal genetic effects do not contribute prenatally to offspring phenotypes. | For most offspring phenotypes, paternal genetic effects are likely to be small or absent, and far smaller in magnitude than maternal genetic effects. | The presence of unmodelled prenatal paternal genetic effects would increase the observed PRS-phenotype covariance in adopted individuals, and consequently inflate estimates of prenatal maternal genetic effects in our SEM. | If paternal genotypes were available from the biological fathers of adopted individuals, the SEM could be extended to estimate prenatal paternal genetic effects as well as the other parameters currently in the model. |
| Adopted individuals do not maintain contact with their biological parents/relatives. |  | Contact between adopted individuals and their biological parents (or other biological relatives) after adoption could impact an adopted individual’s phenotype and increase the covariance between their PRS and phenotype. This would artificially inflate estimates of prenatal maternal genetic effects. | Detailed information on adoptions could be used to exclude these individuals from analyses or attempt to model their presence statistically. |
| Adoptive families are the same as non-adoptive families. | Adopted individuals and adoptive families differ in several respects to the general population. Violation of this assumption is likely to exert more serious consequences on traditional adoption studies which model the phenotypic correlation between biological and adopted relatives. | Our SEM takes into account differences in the offspring phenotypic variance in adoptive and non-adoptive families. More important is whether there is heterogeneity in genetic effects between the two sorts of families (see below). | The presence of adopted individuals with genotyped parents (i.e. both biological and adoptive parents) would allow investigation of whether genetic effects are homogeneous across adoptive and non-adoptive families (see below). |
| Any covariance between the genetic scores in spouses is the same in biological and adoptive families / assortative mating is the same in adoptive and non-adoptive families. | The empirical covariance between PRS in mothers and fathers is small for most phenotypes. Any heterogeneity between adoptive and non-adoptive families may also be small. | Small differences will likely have a modest impact on model parameters. | The availability of genotypes for adopted individuals’ parents (i.e. biological and adoptive parents) would enable testing and relaxation of this assumption. |
| Adopted singletons are unrelated to their adoptive parents. |  | The presence of adoptive parents who are genetically related to their adopted offspring could in some cases increase the type 1 error rate to detect prenatal maternal genetic effects and bias estimates of prenatal and postnatal maternal genetic effects when these relationships are not accurately modelled in the SEM. In general, the effect of including unmodelled related adoptive and biological parents in the SEM depends on whether the adoptive mother or adoptive father was related to the biological parents (i.e. it does not matter whether the adoptive parents were related to the biological mother or father) and the degree of relatedness (i.e. closer relationships had the potential to produce greater bias and type I error rates). | If robust information on the biological relatedness of adopted individuals and their adopted parents can be acquired, then this information can be modelled in the SEM, and asymptotically unbiased estimates of model parameters can be obtained. Even better, genetic information on adopted individuals’ biological and adoptive parents would ensure correct modelling, and would be highly advantageous in terms of statistical power. |
| 1. Limitations/Assumptions Specific to the SEM (these limitations will be relevant to all adoption studies not just UK Biobank) | | | |
| No assortative mating (Positive assortment). | Assortment is likely to be present for educational attainment, but will be less relevant for phenotypes such as birthweight. Our SEM includes a free parameter to model the correlation between maternal and paternal PRS and allows for inflated variance of the offspring PRS. | The effect of assortment on model parameters is likely to be small. | It is possible that our basic SEM could be extended to incorporate the effect of assortment, but would likely require the genotyped parents (biological and adoptive) of adopted offspring. |
| No population stratification. |  | Assuming that population stratification has the effect of biasing estimates of genetic association away from the null, we would expect its presence to increase evidence for prenatal and postnatal maternal genetic effects in our SEM. | Population stratification is typically well controlled through the estimation and inclusion of ancestry informative principal components as covariates in GWAS. Principal components could be estimated and statistically removed from the offspring phenotype before inclusion in our SEM. |
| Adopted individuals were raised in one adoptive family. |  | This assumption is only relevant if genotyped adoptive parents are included in the model, in which case the presence of these individuals could decrease estimates of paternal genetic and postnatal maternal genetic effects. | Detailed information on adoptions could be used to identify and exclude these instances. |
| The total maternal genetic effect can be decomposed into the sum of prenatal and postnatal components. | Additivity is often assumed in statistical models and even if not strictly correct can often yield useful first order approximations. | The effect on the model will depend on the exact form of the underlying relationship. |  |
| Genetic effects are homogenous between biological and adoptive families. | Another way of saying, no genotype x environment interaction. | Heterogeneity in genetic effects implies that it is incorrect to equate path coefficients across adopted and non-adopted individuals. | The presence of adopted individuals with genotyped parents (i.e. both biological and adoptive parents) would allow investigation of whether genetic effects are homogeneous across adoptive and non-adoptive families. For example, if genotype information were present on adopted individuals’ biological and adoptive parents, it would be possible to test whether paternal genetic effects and offspring genetic effects were homogenous across adopted and non-adopted individuals. Note that only homogeneity in total maternal genetic effects (i.e. prenatal + postnatal effects) across adopted and non-adopted individuals could be tested using this design as it is impossible to partition maternal genetic effects into prenatal and postnatal sources in non-adoptive individuals without making the homogeneity assumption. |
| Adopted individuals are placed randomly within the population (including with individuals not genetically related to themselves). | It is possible that adopted children are placed with adoptive parents as similar as possible to the child’s own biological parents. | This may induce similar effects as to being placed with a biological relative (i.e. increased prenatal maternal genetic effects). | Genotype information on adopted individuals’ biological and adoptive parents could be used to test for and model any concerns regarding genetic similarity between adopted children and their adoptive parents. |
| Adoption happened soon after birth. |  | The presence of adopted individuals where the adoption occurred later in life may inflate estimates of prenatal maternal genetic effects (i.e. through contamination by postnatal maternal and paternal genetic effects inflating the PRS-phenotype covariance in adopted singleton individuals). | Detailed information on adoptions could be used to exclude these individuals from analyses or statistically model the age at which they were adopted and the effect on model parameters. |
| Prenatal environmental factors are uncorrelated with maternal PRS. | This gene-environment correlation could arise through active or evocative mechanisms in mothers. | The effect on our SEM would depend on the direction (i.e. positive or negative) of the gene-environment correlation. For example, a positive gene-environment correlation would increase the covariance between offspring PRS and phenotype (in adopted and non-adopted individuals) and bias estimates of the prenatal maternal genetic effect upwards. | Gene-environment correlations are difficult to accommodate in statistical genetics models in general. Maternal PRS could be tested for association with prenatal environmental factors. A significant association would suggest that the assumption of no correlation had been violated (although an absence of association does not imply that the assumption has been proven, since there are other unmeasured environmental factors that may still be correlated with maternal PRS). |
| No evocative (postnatal) gene-environment correlation. | In other words, we assume that the environment that parents provide their children (i.e. which also affects the offspring phenotype under study) is not a consequence of them reacting to their child’s (PRS associated) behavior. | The effect of violating this assumption on our SEM would depend on the direction (i.e. positive or negative) of the gene-environment correlation and whether, both or only one parent were involved. For example, a positive evocative (maternal) gene-environment correlation would increase the covariance between offspring PRS and phenotype in non-adopted individuals only and bias estimates of the postnatal maternal genetic effect upwards. A positive evocative (paternal) gene-environment correlation should inflate estimates of the paternal genetic effect. | Gene-environment correlations are difficult to accommodate in statistical genetics models in general. Child/Parental PRS could be tested for association with postnatal environmental factors. A significant association would suggest that the assumption of no evocative gene-environment correlation had been violated (although an absence of association does not imply that the assumption has been proven, since there are other unmeasured environmental factors that may still be correlated with maternal PRS). |
