# Supplementary material for: Using adopted individuals to partition indirect maternal genetic effects into prenatal and postnatal effects on offspring phenotypes
Source: eLife. 2022 Jul 13;11:e73671. doi: 10.7554/eLife.73671 (PMC9323003; doi:10.7554/eLife.73671)
Supplement: Source code 1. [file elife-73671-code1.zip › 07-09-2021-RA-eLife-73671/Source Code File.docx]

**Source Code File**

# This is code to partition maternal genetic effects into pre- and postnatal effects from Hwang et al. (2021) Using adopted individuals to partition maternal genetic effects into prenatal and postnatal effects on offspring phenotypes

# Please contact Liang-Dar (Daniel) Hwang <d.hwang@uq.edu.au> or David Evans <d.evans1@uq.edu.au> for questions or if you want to fit a more complicated model, e.g., where biological and adoptive parents are related.

# Input data: variance-covariance matrices between offspring phenotype and their own and their relatives' genotypes (or polygenic risk scores) from 7 different family structures.

#Fictitious data used in this example!

# Required R package: "OpenMx"

# Clear global environmental variables

rm(list=ls())

# Load the R package "OpenMx"

library(OpenMx)

# Read in a 4x4 variance-covariance matrix for G1: Biological Parent-offspring trios

# The diagonal contains the variances offspring genotype (Zo), maternal genotype (Zm), paternal genotype (Zp), and offspring phenotype (Y), and the off-diagonal contains their covariances.

group_1 <- matrix(c(1.00, 0.00, 0.50, 0.25, 0.00, 1.00, 0.50, 0.15, 0.50, 0.50, 1.00, 0.25, 0.25, 0.15, 0.25, 1.00), nrow=4, byrow=TRUE) # This is where to read in the variance-covariance matrix

rownames(group_1)<- c("Zm", "Zp", "Zo", "Y")

colnames(group_1)<- c("Zm", "Zp", "Zo", "Y")

N_G1 <- 1000 # This is the number of biological parent-offspring trios used to derive the variance-covariance matrix

# Read in a 3x3 variance-covariance matrix for G2: Mother-offspring pairs

group_2 <- matrix(c(1.00, 0.50, 0.25, 0.50, 1.00, 0.25, 0.25, 0.25, 1.00), nrow=3, byrow=TRUE) # This is where to read in the variance-covariance matrix

rownames(group_2)<- c("Zm", "Zo", "Y")

colnames(group_2)<- c("Zm", "Zo", "Y")

N_G2 <- 4000 # This is the number of biological Mother-offspring pairs used to derive the variance-covariance matrix

# Read in a 3x3 variance-covariance matrix for G3: Father-offspring pairs

group_3 <- matrix(c(1.00, 0.50, 0.15, 0.50, 1.00, 0.25, 0.15, 0.25, 1.00), nrow=3, byrow=TRUE) # This is where to read in the variance-covariance matrix

rownames(group_3)<- c("Zp", "Zo", "Y")

colnames(group_3)<- c("Zp", "Zo", "Y")

N_G3 <- 1800 # This is the number of Father-offspring pairs used to derive the variance-covariance matrix

# Read in a 2x2 variance-covariance matrix for G4: Singletons with Biological Parents

group_4 <- matrix(c(1.00, 0.25, 0.25, 1.00), nrow=2, byrow=TRUE) # This is where to read in the variance-covariance matrix

rownames(group_4)<- c("Zo", "Y")

colnames(group_4)<- c("Zo", "Y")

N_G4 <- 300000 # This is the number of Singletons with Biological Parents used to derive the variance-covariance matrix

# Read in a 2x2 variance-covariance matrix for G5: Singletons with Adoptive Parents

# Zo is adopted individual's genotype and Y is their phenotype

group_5 <- matrix(c(1.00, 0.15, 0.15, 0.96), nrow=2, byrow=TRUE) # This is where to read in the variance-covariance matrix

rownames(group_5)<- c("Zo", "Y")

colnames(group_5)<- c("Zo", "Y")

N_G5 <- 6000 # This is the number of Singletons with Adoptive Parents used to derive the variance-covariance matrix

# Read in a 3x3 variance-covariance matrix for G6: Adoptive Mother - Adopted Child Pairs

# Zo is adopted child's genotype. Zmf is adoptive mother's genotype. Y is adopted child's phenotype.

group_6 <- matrix(c(1.00, 0.0, 0.15, 0.00, 1.0, 0.10, 0.15, 0.1, 0.96), nrow=3, byrow=TRUE) # This is where to read in the variance-covariance matrix

rownames(group_6)<- c("Zo", "Zmf", "Y")

colnames(group_6)<- c("Zo", "Zmf", "Y")

N_G6 <- 0 # This is the number of Adoptive Mother - Adopted Child Pairs used to derive the variance-covariance matrix

# Read in a 3x3 variance-covariance matrix for G7: Biological Mother - Adopted Child Pairs

# Zo is adopted child's genotype. Zmb is biological mother's genotype. Y is adopted child's phenotype.

group_7 <- matrix(c(1.00, 0.50, 0.15, 0.50, 1.00, 0.15, 0.15, 0.15, 0.96), nrow=3, byrow=TRUE) # This is where to read in the variance-covariance matrix

rownames(group_7)<- c("Zo", "Zmb", "Y")

colnames(group_7)<- c("Zo", "Zmb", "Y")

N_G7 <- 50 # This is the number of Biological Mother - Adopted Child Pairs used to derive the variance-covariance matrix

# Model parameters

V <- mxMatrix(type="Full", nrow=1, ncol=1, free=TRUE, values=1, label="v", name="V") #Variance of a SNP or a polygenic risk score

B_OY <- mxMatrix(type="Full", nrow=1, ncol=1, free=TRUE, values=0, label="b_oy", name="B_OY") #Offspring genetic effect

B_MY <- mxMatrix(type="Full", nrow=1, ncol=1, free=TRUE, values=0, label="b_my", name="B_MY") #Post-natal maternal genetic effect

B_PY <- mxMatrix(type="Full", nrow=1, ncol=1, free=TRUE, values=0, label="b_py", name="B_PY") #Paternal genetic effect

G_MY <- mxMatrix(type="Full", nrow=1, ncol=1, free=TRUE, values=0, label="g_my", name="G_MY") #Pre-natal maternal genetic effect

E1 <- mxMatrix(type="Full", nrow=1, ncol=1, free=TRUE, values=0.9, label="e1", name="E1") #Phenotypic error variance in biological families

E2 <- mxMatrix(type="Full", nrow=1, ncol=1, free=TRUE, values=0.9, label="e2", name="E2") #Phenotypic error variance in adopted families

R <- mxMatrix(type="Full", nrow=1, ncol=1, free=TRUE, values=0, label="rho", name="R") #Covariance between maternal and paternal genotypes

# Define elements of expected covariance matrix in terms of parameters

# G1 Parent offspring trios

c11_G1 <- mxAlgebra(expression=V, name="C11_G1")

c12_G1 <- mxAlgebra(expression=R, name="C12_G1")

c13_G1 <- mxAlgebra(expression=0.5*V+0.5*R, name="C13_G1")

c14_G1 <- mxAlgebra(expression=(G_MY + B_MY)*V + 0.5*B_OY*V + B_PY*R + 0.5*B_OY*R, name="C14_G1")

c21_G1 <- mxAlgebra(expression=R, name="C21_G1")

c22_G1 <- mxAlgebra(expression=V, name="C22_G1")

c23_G1 <- mxAlgebra(expression=0.5*V+0.5*R, name="C23_G1")

c24_G1 <- mxAlgebra(expression=B_PY*V + 0.5*B_OY*V + (G_MY+B_MY)*R + 0.5*B_OY*R, name="C24_G1")

c31_G1 <- mxAlgebra(expression=0.5*V+0.5*R, name="C31_G1")

c32_G1 <- mxAlgebra(expression=0.5*V+0.5*R, name="C32_G1")

c33_G1 <- mxAlgebra(expression=V+0.5*R, name="C33_G1")

c34_G1 <- mxAlgebra(expression=B_OY*(V+0.5*R) + 0.5*V*(G_MY + B_MY) + 0.5*V*B_PY + 0.5*(G_MY + B_MY)*R + 0.5*B_PY*R, name="C34_G1")

c41_G1 <- mxAlgebra(expression=(G_MY + B_MY)*V + 0.5*B_OY*V + B_PY*R + 0.5*B_OY*R, name="C41_G1")

c42_G1 <- mxAlgebra(expression=B_PY*V + 0.5*B_OY*V + (G_MY+B_MY)*R + 0.5*B_OY*R, name="C42_G1")

c43_G1 <- mxAlgebra(expression=B_OY*(V+0.5*R) + 0.5*V*(G_MY + B_MY) + 0.5*V*B_PY + 0.5*(G_MY+B_MY)*R + 0.5*B_PY*R, name="C43_G1")

c44_G1 <- mxAlgebra(expression=B_PY^2*V + B_OY^2*(V+0.5*R) + (G_MY + B_MY)^2*V + B_OY*V*(G_MY + B_MY) + B_OY*V*B_PY + E1 + 2*B_PY*R*(G_MY+B_MY) + B_PY*B_OY*R + B_OY*(G_MY+B_MY)*R, name="C44_G1")

# G2 Mother offspring pairs

c11_G2 <- mxAlgebra(expression=V, name="C11_G2")

c12_G2 <- mxAlgebra(expression=0.5*V+0.5*R, name="C12_G2")

c13_G2 <- mxAlgebra(expression=(G_MY + B_MY)*V + 0.5*B_OY*V + B_PY*R + 0.5*B_OY*R, name="C13_G2")

c21_G2 <- mxAlgebra(expression=0.5*V+0.5*R, name="C21_G2")

c22_G2 <- mxAlgebra(expression=V+0.5*R, name="C22_G2")

c23_G2 <- mxAlgebra(expression=B_OY*(V+0.5*R) + 0.5*V*(G_MY + B_MY) + 0.5*V*B_PY + 0.5*(G_MY + B_MY)*R + 0.5*B_PY*R, name="C23_G2")

c31_G2 <- mxAlgebra(expression=(G_MY + B_MY)*V + 0.5*B_OY*V + B_PY*R + 0.5*B_OY*R, name="C31_G2")

c32_G2 <- mxAlgebra(expression=B_OY*(V+0.5*R) + 0.5*V*(G_MY + B_MY) + 0.5*V*B_PY + 0.5*(G_MY+B_MY)*R + 0.5*B_PY*R, name="C32_G2")

c33_G2 <- mxAlgebra(expression=B_PY^2*V + B_OY^2*(V+0.5*R) + (G_MY + B_MY)^2*V + B_OY*V*(G_MY + B_MY) + B_OY*V*B_PY + E1 + 2*B_PY*R*(G_MY+B_MY) + B_PY*B_OY*R + B_OY*(G_MY+B_MY)*R, name="C33_G2")

# G3 Father offspring pairs

c11_G3 <- mxAlgebra(expression=V, name="C11_G3")

c12_G3 <- mxAlgebra(expression=0.5*V+0.5*R, name="C12_G3")

c13_G3 <- mxAlgebra(expression=B_PY*V + 0.5*B_OY*V + (G_MY+B_MY)*R + 0.5*B_OY*R, name="C13_G3")

c21_G3 <- mxAlgebra(expression=0.5*V+0.5*R, name="C21_G3")

c22_G3 <- mxAlgebra(expression=V+0.5*R, name="C22_G3")

c23_G3 <- mxAlgebra(expression=B_OY*(V+0.5*R) + 0.5*V*(G_MY + B_MY) + 0.5*V*B_PY + 0.5*(G_MY + B_MY)*R + 0.5*B_PY*R, name="C23_G3")

c31_G3 <- mxAlgebra(expression=B_PY*V + 0.5*B_OY*V + (G_MY+B_MY)*R + 0.5*B_OY*R, name="C31_G3")

c32_G3 <- mxAlgebra(expression=B_OY*(V+0.5*R) + 0.5*V*(G_MY + B_MY) + 0.5*V*B_PY + 0.5*(G_MY+B_MY)*R + 0.5*B_PY*R, name="C32_G3")

c33_G3 <- mxAlgebra(expression=B_PY^2*V + B_OY^2*(V+0.5*R) + (G_MY + B_MY)^2*V + B_OY*V*(G_MY + B_MY) + B_OY*V*B_PY + E1 + 2*B_PY*R*(G_MY+B_MY) + B_PY*B_OY*R + B_OY*(G_MY+B_MY)*R, name="C33_G3")

# G4 Singletons (biological)

c11_G4 <- mxAlgebra(expression=V+0.5*R, name="C11_G4")

c12_G4 <- mxAlgebra(expression=B_OY*(V+0.5*R) + 0.5*V*(G_MY + B_MY) + 0.5*V*B_PY + 0.5*(G_MY + B_MY)*R + 0.5*B_PY*R, name="C12_G4")

c21_G4 <- mxAlgebra(expression=B_OY*(V+0.5*R) + 0.5*V*(G_MY + B_MY) + 0.5*V*B_PY + 0.5*(G_MY+B_MY)*R + 0.5*B_PY*R, name="C21_G4")

c22_G4 <- mxAlgebra(expression=B_PY^2*V + B_OY^2*(V+0.5*R) + (G_MY + B_MY)^2*V + B_OY*V*(G_MY + B_MY) + B_OY*V*B_PY + E1 + 2*B_PY*R*(G_MY+B_MY) + B_PY*B_OY*R + B_OY*(G_MY+B_MY)*R, name="C22_G4")

# G5 Singletons (adopted)

c11_G5 <- mxAlgebra(expression=V+0.5*R, name="C11_G5")

c12_G5 <- mxAlgebra(expression=B_OY*(V+0.5*R) + 0.5*V*G_MY + 0.5*G_MY*R, name="C12_G5")

c21_G5 <- mxAlgebra(expression=B_OY*(V+0.5*R) + 0.5*V*G_MY + 0.5*G_MY*R, name="C21_G5")

c22_G5 <- mxAlgebra(expression=B_OY^2*(V+0.5*R) + G_MY^2*V + B_MY^2*V + B_PY^2*V + B_OY*V*G_MY + B_OY*G_MY*R + E2 + 2*B_MY*B_PY*R, name="C22_G5")

# G6 Foster Mother - Adopted Child Pairs

c11_G6 <- mxAlgebra(expression=V+0.5*R, name="C11_G6")

c12_G6 <- mxAlgebra(expression=0, name="C12_G6")

c13_G6 <- mxAlgebra(expression=B_OY*(V+0.5*R) + 0.5*V*G_MY + 0.5*G_MY*R, name="C13_G6")

c21_G6 <- mxAlgebra(expression=0, name="C21_G6")

c22_G6 <- mxAlgebra(expression=V, name="C22_G6")

c23_G6 <- mxAlgebra(expression=B_MY*V, name="C23_G6")

c31_G6 <- mxAlgebra(expression=B_OY*(V+0.5*R) + 0.5*V*G_MY + 0.5*G_MY*R, name="C31_G6")

c32_G6 <- mxAlgebra(expression=B_MY*V, name="C32_G6")

c33_G6 <- mxAlgebra(expression=B_OY^2*(V+0.5*R) + G_MY^2*V + B_MY^2*V + B_PY^2*V + B_OY*V*G_MY + B_OY*G_MY*R + E2 + 2*B_MY*B_PY*R, name="C33_G6")

# G7 Biological Mother - Adopted Child Pairs

c11_G7 <- mxAlgebra(expression=V+0.5*R, name="C11_G7")

c12_G7 <- mxAlgebra(expression=0.5*V+0.5*R, name="C12_G7")

c13_G7 <- mxAlgebra(expression=B_OY*(V+0.5*R) + 0.5*V*G_MY + 0.5*G_MY*R, name="C13_G7")

c21_G7 <- mxAlgebra(expression=0.5*V+0.5*R, name="C21_G7")

c22_G7 <- mxAlgebra(expression=V, name="C22_G7")

c23_G7 <- mxAlgebra(expression=G_MY*V + 0.5*B_OY*V + 0.5*B_OY*R, name="C23_G7")

c31_G7 <- mxAlgebra(expression=B_OY*(V+0.5*R) + 0.5*V*G_MY + 0.5*G_MY*R, name="C31_G7")

c32_G7 <- mxAlgebra(expression=G_MY*V + 0.5*B_OY*V + 0.5*B_OY*R, name="C32_G7")

c33_G7 <- mxAlgebra(expression=B_OY^2*(V+0.5*R) + G_MY^2*V + B_MY^2*V + B_PY^2*V + B_OY*V*G_MY + B_OY*G_MY*R + E2 + 2*B_MY*B_PY*R, name="C33_G7")

# Expected covariance matrices

expCov_G1 <- mxAlgebra( expression= rbind( cbind(C11_G1, C12_G1, C13_G1, C14_G1), cbind(C21_G1, C22_G1, C23_G1, C24_G1), cbind(C31_G1, C32_G1, C33_G1, C34_G1), cbind(C41_G1, C42_G1, C43_G1, C44_G1)), name="expCov_G1")

expCov_G2 <- mxAlgebra( expression= rbind( cbind(C11_G2, C12_G2, C13_G2), cbind(C21_G2, C22_G2, C23_G2), cbind(C31_G2, C32_G2, C33_G2)), name="expCov_G2")

expCov_G3 <- mxAlgebra( expression= rbind( cbind(C11_G3, C12_G3, C13_G3), cbind(C21_G3, C22_G3, C23_G3), cbind(C31_G3, C32_G3, C33_G3)), name="expCov_G3")

expCov_G4 <- mxAlgebra( expression= rbind( cbind(C11_G4, C12_G4), cbind(C21_G4, C22_G4)), name="expCov_G4")

expCov_G5 <- mxAlgebra( expression= rbind( cbind(C11_G5, C12_G5), cbind(C21_G5, C22_G5)), name="expCov_G5")

expCov_G6 <- mxAlgebra( expression= rbind( cbind(C11_G6, C12_G6, C13_G6), cbind(C21_G6, C22_G6, C23_G6), cbind(C31_G6, C32_G6, C33_G6)), name="expCov_G6")

expCov_G7 <- mxAlgebra( expression= rbind( cbind(C11_G7, C12_G7, C13_G7), cbind(C21_G7, C22_G7, C23_G7), cbind(C31_G7, C32_G7, C33_G7)), name="expCov_G7")

# Create Data Objects for Multiple Groups

dataG1 <- mxData(observed=(group_1), type="cov", numObs=N_G1)

dataG2 <- mxData(observed=(group_2), type="cov", numObs=N_G2)

dataG3 <- mxData(observed=(group_3), type="cov", numObs=N_G3)

dataG4 <- mxData(observed=(group_4), type="cov", numObs=N_G4)

dataG5 <- mxData(observed=(group_5), type="cov", numObs=N_G5)

dataG6 <- mxData(observed=(group_6), type="cov", numObs=N_G6)

dataG7 <- mxData(observed=(group_7), type="cov", numObs=N_G7)

# Create Expectation Objects for Multiple Groups

expG1 <- mxExpectationNormal( covariance="expCov_G1", dimnames=c("Zm","Zp","Zo","Y") )

expG2 <- mxExpectationNormal( covariance="expCov_G2", dimnames=c("Zm","Zo", "Y") )

expG3 <- mxExpectationNormal( covariance="expCov_G3", dimnames=c("Zp","Zo","Y") )

expG4 <- mxExpectationNormal( covariance="expCov_G4", dimnames=c("Zo","Y") )

expG5 <- mxExpectationNormal( covariance="expCov_G5", dimnames=c("Zo","Y") )

expG6 <- mxExpectationNormal( covariance="expCov_G6", dimnames=c("Zo","Zmf","Y") )

expG7 <- mxExpectationNormal( covariance="expCov_G7", dimnames=c("Zo","Zmb","Y") )

funML <- mxFitFunctionML()

# Create Model Objects for Multiple Groups

pars <- list( V, B_OY, B_MY, B_PY, G_MY, E1, E2, R)

modelG1 <- mxModel( V, B_OY, B_MY, B_PY, G_MY, E1, R, c11_G1, c12_G1, c13_G1, c14_G1, c21_G1, c22_G1, c23_G1, c24_G1, c31_G1, c32_G1, c33_G1, c34_G1, c41_G1, c42_G1, c43_G1, c44_G1, expG1, expCov_G1, dataG1, funML, name="G1" )

modelG2 <- mxModel( V, B_OY, B_MY, B_PY, G_MY, E1, R, c11_G2, c12_G2, c13_G2, c21_G2, c22_G2, c23_G2, c31_G2, c32_G2, c33_G2, expG2, expCov_G2, dataG2, funML, name="G2" )

modelG3 <- mxModel( V, B_OY, B_MY, B_PY, G_MY, E1, R, c11_G3, c12_G3, c13_G3, c21_G3, c22_G3, c23_G3, c31_G3, c32_G3, c33_G3, expG3, expCov_G3, dataG3, funML, name="G3" )

modelG4 <- mxModel( V, B_OY, B_MY, B_PY, G_MY, E1, R, c11_G4, c12_G4, c21_G4, c22_G4, expG4, expCov_G4, dataG4, funML, name="G4" )

modelG5 <- mxModel( V, B_OY, B_MY, B_PY, G_MY, E2, R, c11_G5, c12_G5, c21_G5, c22_G5, expG5, expCov_G5, dataG5, funML, name="G5" )

modelG6 <- mxModel( V, B_OY, B_MY, B_PY, G_MY, E2, R, c11_G6, c12_G6, c13_G6, c21_G6, c22_G6, c23_G6, c31_G6, c32_G6, c33_G6, expG6, expCov_G6, dataG6, funML, name="G6" )

modelG7 <- mxModel( V, B_OY, B_MY, B_PY, G_MY, E2, R, c11_G7, c12_G7, c13_G7, c21_G7, c22_G7, c23_G7, c31_G7, c32_G7, c33_G7, expG7, expCov_G7, dataG7, funML, name="G7" )

multi <- mxFitFunctionMultigroup( c("G1","G2","G3","G4","G5","G6","G7") )

modelFull <- mxModel( "Full_Model", pars, modelG1, modelG2, modelG3, modelG4, modelG5, modelG6, modelG7, multi)

# RUN MODEL

fitFull <- mxRun( modelFull, intervals=F )

sumFull <- summary( fitFull )

sumFull

# v, variance of the SNP (polygenic risk score)

# B_OY, offspring genetic effect

# b_my, postnatal maternal genetic effect

# b_py, paternal genetic effect

# g_my, prenatal maternal genetic effect

# e1, error variance of biological children's phenotype

# e2, error variance of adopted children's phenotype

# rho, covariance between maternal and paternal genotypes
